# Investigating the modulatory effects of *Moringa oleifera* on the gut microbiota of chicken model through metagenomic approach

**DOI:** 10.3389/fvets.2023.1153769

**Published:** 2023-06-01

**Authors:** Sowmiya Soundararajan, Jasmine Selvakumar, Zion Mercy Maria Joseph, Yuvapriya Gopinath, Vaishali Saravanan, Rameshkumar Santhanam

**Affiliations:** ^1^Department of Biotechnology and Bioinformatics, Bishop Heber College (Autonomous), Affiliated With Bharathidasan University, Tiruchirappalli, Tamil Nadu, India; ^2^Faculty of Science and Marine Environment, Universiti Malaysia Terengganu, Kuala Nerus, Terengganu, Malaysia

**Keywords:** phytobiotics, *Moringa oleifera*, gut microbiota, metagenomics, cytotoxic, chicken, cecum, *Enterococcus faecium*

## Abstract

**Introduction:**

This study aimed to assess the effects of supplementing chicken feed with *Moringa oleifera* leaf powder, a phytobiotic, on the gastrointestinal microbiota. The objective was to examine the microbial changes induced by the supplementation.

**Methods:**

A total of 40, one-day-old chickens were fed their basal diet for 42 days and then divided into two groups: SG1 (basal diet) and SG2 (basal diet + 10 g/kg *Moringa oleifera* leaf powder). Metagenomics analysis was conducted to analyze operational taxonomic units (OTUs), species annotation, and biodiversity. Additionally, 16S rRNA sequencing was performed for molecular characterization of isolated gut bacteria, identified as *Enterococcus faecium*. The isolated bacteria were tested for essential metabolites, demonstrating antibacterial, antioxidant, and anticancer activities.

**Results and discussion:**

The analysis revealed variations in the microbial composition between the control group (SG1) and the *M. oleifera*-treated group (SG2). SG2 showed a 47% increase in Bacteroides and a 30% decrease in Firmicutes, Proteobacteria, Actinobacteria, and Tenericutes compared to SG1. TM7 bacteria were observed exclusively in the *M. oleifera*-treated group. These findings suggest that *Moringa oleifera* leaf powder acts as a modulator that enhances chicken gut microbiota, promoting the colonization of beneficial bacteria. PICRUSt analysis supported these findings, showing increased carbohydrate and lipid metabolism in the *M.oleifera*-treated gut microbiota.

**Conclusion:**

This study indicates that supplementing chicken feed with *Moringa oleifera* leaf powder as a phytobiotic enhances the gut microbiota in chicken models, potentially improving overall health. The observed changes in bacterial composition, increased presence of Bacteroides, and exclusive presence of TM7 bacteria suggest a positive modulation of microbial balance. The essential metabolites from isolated *Enterococcus faecium* bacteria further support the potential benefits of *Moringa oleifera* supplementation.

## 1. Introduction

The stability and composition of the intestinal microbiota play a significant role in ensuring healthy gut function. Several factors modulate gut microbiota, which, in turn, impacts an individual's health status (1). Unbalanced and unhealthy diets, as well as infections, can disrupt the balance of the gut microbiota, leading to dysbiosis and negatively affecting human health (2). This research aimed to employ metagenomics to evaluate the implications of phytobiotics in improving the gut microbial ecosystem in chicken models. The human intestine harbors both beneficial and pathogenic microbes, which normally coexist in a delicate balance (3). Maintaining a proper microbial balance between beneficial and harmful organisms in the gut is widely recognized as crucial for promoting good gut health and maintaining overall wellbeing. Normally, the gut contains a higher proportion of beneficial or probiotic bacteria, making up ~80% of the microbial population, with the remaining 20% consisting of pathogens. Any changes to this balance are known to disrupt the gut microbiome (4). It has been reported that probiotic bacteria aid in the digestion of complex nutrients and produce crucial dietary compounds with therapeutic benefits (5). In a study on mammals, it has been reported that probiotic bacteria aid in regulating digestion by facilitating bile acid synthesis (6), assimilating fatty acids and proteins, synthesizing antioxidants, and producing SCFAs (7). Achieving a balanced intestinal flora is crucial for improving the gut health of chickens, leading to enhanced growth and overall performance (8, 9).

Nutraceuticals, which are natural products used to treat various health conditions in humans, have been shown to have potential benefits in modulating the gut microbiota and the immune system (10, 11). These products have been used to treat a range of ailments, such as carcinoma, metabolic disorders, osteopenia, and anxiety. The potential use of nutraceuticals for managing and preventing enteric infections in chickens is an area that deserves scholarly attention (12). Additionally, the ability of nutraceuticals to improve gut morphology and nutrient absorption (13) may encourage nutritionists to incorporate these supplements into chicken diets for the production performance of improved poultry. Phytobiotics are natural compounds that are derived from plants and added to animal feed to improve the health and wellbeing of animals (14). Plants are known to exhibit several therapeutic properties owing to the production of an array of phytochemicals such as terpenoids (mono- and sesquiterpenes, growth hormones, etc.), polyphenolic compounds (tannic acid), glucosinolates, and naturally occurring substances (present as alcohols, aldehydes, ketones, esters, ethers, lactones, etc.) (15). In animal models, the potential benefits of phytobiotics may involve modulating the frequency of biological membranes in the microbiota, which results in membrane damage to pathogenic organisms, increasing the hydrophobicity of the bacterial population, which can also affect the surface morphology of microbes and, in turn, impact the pathogenicity properties of these organisms (16). Phytobiotics may also enhance the growth of beneficial bacteria such as *Lactobacilli* sp. and *Bifidobacteria* sp. in the intestines, function as immunomodulating agents, and protect the gut muscle from microbes (17).

Phytobiotics have been observed to modulate the intestinal microbiota, favorably colonize specific sites in the gut, and produce metabolites that exhibit antagonistic properties against pathogenic organisms that produce toxins (18). Moreover, they have been reported to boost host–microbe interactions, enhance the host immune system, and facilitate the growth of a healthy gut microbiome (19). In recent years, various novel approaches have emerged regarding the use of phytobiotics in poultry nutrition, as they are a diverse group of biologically active compounds derived from a variety of plants (20). Phytobiotics as a feed additive are still in the early stages of research and development compared to other antibiotic-based approaches and require further investigation for their potential use (21). *Moringa oleifera*, a plant with medicinal properties, is utilized as a source of feed for both humans and animals, such as chicken feed, due to its high nutritional value (22). *Moringa oleifera* leaf powder (MOLP) is rich in proteins, vitamins A, B, and C, and minerals, including calcium and iron (23). The protein content of *Moringa oleifera* leaves ranges from 21 to 25% of their dry weight, and its protein quality is high (24). The *Moringa* plant is often referred to as the “Miracle Tree” due to its various medicinal benefits, such as its hypocholesterolemic properties (25). Additionally, the carotenoid compounds found in moringa have implications for chicken meat quality and can serve as an alternative to traditional feed ingredients (26). Supplementation with *Moringa oleifera* leaf powder (MOLP) in the diets of broiler chickens has been shown to have positive effects on growth performance, antioxidant status, cecum microbiota modulation, and enteric pathogen prevention without any negative effects (27). Broiler chicken meat is an important source of animal protein for human consumption, with its global demand expected to reach 153.85 metric kilotons by 2031 (28), and the poultry industry has significantly focused on increasing production. Previously, antibiotic growth promoters played a major role in the industry (29); however, there has been a recent shift toward the use of phytobiotics as an alternative to antibiotics (30). Researchers have also been investigating dietary modulations to improve the production of chickens by modulating their intestinal microbiota (31, 32).

Existing studies have revealed that metagenomics is a valuable tool for investigating bacterial populations in their unique habitats (33). Next-generation sequencing has enabled researchers to study complex ecological interactions, such as lateral gene transfer, phage-host dynamics, and metabolic complementation (34, 35). High-throughput comparative metagenomics, powered by advancements in next-generation sequencing (NGS) technologies, has led to an explosion of ongoing research that has greatly improved our understanding of microbial population composition and function in diverse environments (36). Simple and low-cost metagenomic techniques have been used in several studies to comprehend the dynamics of the microbial community (37).

The focus of this study was to enhance the microbiota's quality of health through a diet rich in phytobiotics using *Moringa oleifera*. Due to the similarity between the chicken and human microbiomes, chicken models are highly sought after for human studies. Thus, chickens have been utilized as a model to investigate the therapeutic potential of the intestinal microbiome modulated through MOLP. Consequently, we performed metagenomic-based comparative studies on the gut microbiomes of normal and phytobiotic-treated chickens to determine and understand microbial diversity.

## 2. Materials and methods

### 2.1. Ethics approval for the study

The study was conducted according to the approved procedures after obtaining approval from the Institutional Animal Ethics Committee (IAEC) at Bharathidasan University, Tiruchirappalli, Tamil Nadu, India for its planning and execution. All procedures adhered to applicable rules and regulations. The registration number for the study was 418/GO/Re/S/01/CPCSEA, dt.24.07.2018; (BDU/IAEC/P06/2021).

### 2.2. Plant collection

Fresh green *Moringa oleifera* leaves were collected from Kolli Hills, Namakkal, Tamil Nadu, India, in the month of March. The plant and the specimen voucher were authenticated and deposited (2979) at the Dept. of Botany, St. Joseph's College, Trichy, Tamil Nadu, India. To minimize leaching, the leaves were dried without being exposed to direct sunlight. To prevent fungal development, the leaves were flipped often during the drying process. The dried leaves were then ground into a fine powder (38).

### 2.3. Experimental design for birds

Forty 1-day-old healthy male broiler chicks, each weighing about 50 g, were purchased from the Veterinary College and Research Institute, Namakkal, Tamil Nadu, India. The chicks were split into two different floor pens and provided with unlimited water and two different diets. Each group had 10 replicates, and each chicken was kept under controlled conditions with *ad libitum* access to feed and freshwater around the clock. During the 1st week, the experimental house's temperature and relative humidity (RH) were kept at 35 ± 1°C and 70 ± 5%, respectively. Subsequently, the temperature was reduced by 3°C every week until it reached 26 ± 1°C with an RH of 65 ± 5% on day 21 and was maintained until the study ended on day 42 (39). The first group (SG1) was fed a normal basal diet (NBD) without any phytobiotic supplementation, while the second group was fed a phytobiotic supplement of *Moringa oleifera* leaf powder (MOLP) at a rate of 10 g/kg, along with a starter meal. From day 15 to 42, the starter meal was replaced with a grower-finisher meal (40). The ingredient composition of the diet is provided in Table 1. The phytobiotic-treated group was not administered any antibiotics or vaccinations.

**Table 1:** Ingredient composition of broiler booster and finisher diets formulated with the inclusion of *Moringa oleifera* leaf powder meal (%).

**S. No**	**Ingredients**	**Broiler starter %**		**Broiler finisher %**	
		**SG1**	**SG2**	**SG1**	**SG2**
1	Maize Meal	58.00	57.50	61.00	61.50
2	Fish meal	14.00	14.00	08.10	08.60
3	Soya beans cake	16.00	17.10	11.00	11.50
4	Rice bran	02.50	00.80	09.50	06.50
5	Super concentrate	05.00	05.00	05.00	05.00
6	Lysine	00.10	00.10	00.10	00.10
7	Calcium phosphate	00.30	00.30	00.70	00.69
8	Sunflower oil	01.20	01.20	00.60	00.61
9	Limestone	02.00	02.00	03.50	03.50
10	Sodium chloride	00.25	00.25	00.25	00.25
11	Vit +mineral premix	00.25	00.25	00.25	00.25
12	Moringa leaf powder	-	01.00	-	01.00
	Total	100	100	100	100

Body weight measurements of the chickens were taken every morning for the entire 42 days of the experiment. The feed consumption per group was calculated every day to determine the average daily feed intake (ADFI). Subsequently, the feed conversion rate was recorded by taking data on average daily gain (ADG) and average daily feed intake (ADFI). The basal diet developed by the Nutrient Requirements of Poultry was used to fulfill the poultry's nutritional needs (41). From each group, one healthy chicken was chosen for euthanasia. The chicken was euthanized by bloodletting just outside the neck after aseptic abdominal incisions. The weights were measured after rapid cecum excision, and cecal digesta samples were taken to the lab for further analysis. These samples were immediately frozen and stored in liquid nitrogen (−80°C) (42, 43).

### 2.4. Metagenomic analysis

#### 2.4.1. Sequencing methodology

A standard protocol for DNA extraction for metagenomic analysis was followed, as reported earlier (44). To amplify the 16S rRNA hypervariable region V3–V4, 25 ng of DNA was employed. The response incorporates KAPA HiFi HotStart Ready Mix and a modified ultimate concentration of 100 nm primers 341F and 785R (45) (Supplementary Table 1). The PCR began with a 5-min denaturation at 95°C, followed by 25 cycles of 95°C for 30 s, 55°C for 45 s, and 72°C for 30 s, with a final 7-min extension at 72°C. Ampure beads were used to purify the amplicons by deleting any unneeded primers. Illumina sequencing was used for an additional of 8 PCR cycles. The following barcode adapters were used to create the sequencing libraries.

##### 2.4.1.1. Adapter sequence

P7 adapter read1 AGATCGGAAGAGCACACGTCTGAACTCCAGTCA.

P5 adapter read2 AGATCGGAAGAGCGTCGTGTAGGGAAAGAGTGT.

#### 2.4.2. Sequence data for QC

Sequencing data were generated using Illumina MiSeq. The quality of the data was evaluated using FastQC (46) and MultiQC (47, 48) tools. The quality of the data was assessed based on the distribution of base call quality, with percentage of bases over Q20, Q30, % GC, and sequencing adapter contamination (44).

#### 2.4.3. Sequencing data analysis

To eliminate the degenerate primers, the readings were trimmed (20 bp) from the 5' end (49). Trimgalore was used to eliminate adaptor sequences and low-quality bases from the trimmed reads. The QC-passed reads were imported into Mothur (50), where the pairs were aligned and assembled into contigs. Only contigs with lengths ranging from 300 and 532 bp were kept after being checked for errors. Any contigs with unclear base calls were discarded. The high-quality contigs were examined for duplicate sequences, and duplicates were combined. Although the primers used in the study targeted bacterial 16S rRNA, there was a possibility of non-specific amplification for other areas. To account for this, we aligned the contigs against a reference database of known 16S rRNA sequences. Most contigs would align to their relevant database area, depending on the variable region being amplified. Any unclear contigs that matched other areas in the database were removed. After this procedure, the gaps and overhangs at the ends of the contigs were deleted, and any chimeras that may have occurred due to mistakes were removed. Contigs with chimeric areas were identified using the UCHIME method (51). To detect and exclude any potential chimeric sequences, a known reference of all chimeric sequences was employed. The reference database used in this study was based on the GREEN GENES v. 13.8–99 database (52). The contigs were also classified into operational taxonomic units (OTUs). Following categorization, the abundance of each OTU was estimated. The group abundance was investigated using the alpha diversity estimators Chao1, ACE, Shannon, and Simpson. The analysis of beta diversity using Fisher's exact test was carried out on samples using STAMP to identify statistically significant differences in OTU abundance between the samples (53).

### 2.5. Isolation of probiotic bacteria

The cecum samples of the control group (SG1) and the phytobiotic-treated chicken MOLP group (SG2) were crushed using PBS buffer and homogenized with a mortar and pestle to obtain a fine paste-like consistency for further investigation (44). To obtain countable bacterial isolates, the sample was serially diluted and placed on nutrient and MRS agar plates, which were then cultured in both aerobic and anaerobic environments for 24–48 h. After incubation, the plates were examined for colonies, which were then purified and identified using 16S rRNA sequencing (54).

### 2.6. Isolation of lipids

A centrifuge tube containing 5 ml of culture was centrifuged at 5,000 rpm for 20 min. The upper layer of the centrifuge tube was discarded, and the lower-layer pellet was rinsed twice with double-distilled water. Next, 3 ml of HCl was added to the pellet, and the centrifuge tube was placed in a water bath for 1–1.30 h. Afterward, methanol and chloroform were added in a 1:1 ratio, and the mixture was left undisturbed for 24 h. The resulting supernatant was collected and considered to be lipids. To assess the qualitative status of lipid production in cells, Sudan Black B stain was used for staining, and the samples were observed under a microscope. The color of the stain changed from dark to light blue, as reported by Schittler et al. (55).

### 2.7. Biological assays

#### 2.7.1. Antibacterial activity: disc diffusion method

To evaluate the antimicrobial activity, the metabolites (lipids) obtained from the SG1 and SG2 groups were analyzed using the disc diffusion assay (56). The results were compared to those obtained using standard antibiotics. The bacterial strains used in the study primarily belonged to the *Enterobacteriaceae* family, with *Enterococcus faecium*, a bacterium commonly found in the colon, being the primary focus. Muller Hinton Agar (MHA) was prepared and poured into sterile Petri plates, and different bacterial cultures were inoculated on the MHA plates using a swab. Sterile disks were diffused with ~60 μL of isolated metabolites and placed over Petri plates along with standard antibiotic disks. The plates were incubated at 37°C for 18–24 h to allow zones of inhibition to develop. Additionally, the minimal inhibitory concentration (MIC) was determined using the microbroth dilution method (57).

#### 2.7.2. Antioxidant activity

The metabolites (lipids) produced from *E. faecium*, which were isolated from the cecal digesta, were used for the antioxidant assay. Specifically, the lipids were obtained from both the control group (SG1) and the group of chickens that had been treated with phytobiotics (SG2). The DPPH radical scavenging assay is a commonly used method for evaluating the ability of natural substances to scavenge free radicals and measuring the scavenging capacity of antioxidant compounds against stable radicals. The free radical scavenging ability of the extracts was analyzed *in vitro* using the DPPH radical, following the method by Shimada et al. with slight modifications. In this assay, 1.0 ml of extracts at different doses (0–4.5 μg/mL) were mixed with 1.0 ml of a 0.8 mM DPPH solution. The mixture was vigorously mixed and allowed to stand for 30 min, after which the absorbance was measured at 517 nm against a reagent blank. Ascorbic acid (1 μg/mL) was used as the standard (58). The percentage of scavenging DPPH radical inhibition was calculated using the following formula:


$$\begin{array}{l} \%\;Inhibition=\left[\left(Control-Test\right)/Control\right]\;\times \;100 \end{array}$$

#### 2.7.3. Anti-cancer activity

##### 2.7.3.1. Cell culture and MTT assay

The lipids as metabolites produced from *E. faecium*, which was isolated from the cecal digesta, were used for the MTT assay to assess cytotoxicity. Specifically, the lipids were obtained from both the control group (SG1) and the group of chickens that had been treated with phytobiotics (SG2). HT-29, a human colon cancer cell line, was obtained from NCCS, Pune, cultured in DMEM liquid medium enriched with 10% fetal bovine serum (FBS), 100 g/mL penicillin, and 100 g/mL streptomycin, and maintained at 37°C in a 5% CO2 environment. The SG1 and SG2 samples were analyzed for *in vitro* cytotoxicity using the MTT assay on HT-29 cells. First, trypsinized HT-29 cells were collected and pooled in a 15-mL tube. The cells were then placed on a 96-well tissue culture plate at a density of 1 × 105 cells/mL cells/well (200 μL) in DMEM media with 10% FBS and 1% antibiotic solution for 24–48 h at 37°C. The wells were then rinsed with sterile PBS in a serum-free DMEM medium and treated with different concentrations (0–500 μg/mL) of both samples. Each sample was duplicated three times, and the cells were cultured for 24 h at 37°C in a humidified 5% CO_2_ incubator. After incubation, MTT (20 μL of 5 mg/mL) was added to each well, and the cells were cultivated for another 2–4 h until purple precipitates were easily visible underneath an inverted microscope. The medium and MTT (220 μL) were then aspirated from the wells and rinsed with 1X PBS (200 μL). To disperse the formazan crystals, 100 μL of DMSO was added, and the plate was agitated for 5 min. The absorbance for each well was measured at 570 nm using a microplate reader (Thermo Fisher Scientific, USA), and the percentage cell viability and IC50 value were estimated using GraphPad Prism 6.0 software (USA) (59).


$$\begin{array}{l} Formula:\;Cell\;viability\;\%=Test\;OD/Control\;OD\;X\;100 \end{array}$$

## 3. Results

### 3.1. Sample collection, sequencing, and quality check

The cecal of chickens from both the control and MOLP-treated groups (SG1, SG2) were aseptically extracted and subjected to genomic DNA extraction (Supplementary Figures 1, 2). The V4 hyper-variable region of the 16S rRNA gene was then amplified and sequenced using Illumina MiSeq. The sequencing data underwent quality checks using FastQC and MultiQC software to ensure base call quality scores of at least 98.50%, sequence quality scores above Q20 and Q30, 52.5% GC, and no sequencing adapter contamination (Supplementary Table 2). All samples met the QC threshold with a Q20 score of >95%. Quality scores were graphed on the y-axis (Supplementary Figure 3), with higher scores indicating better base call quality. The samples were sequenced in a single FastQ format and deposited in the NCBI-SRA portal (Accession No.: PRJNA823890, SAMN27363940, and SAMN27363941). Histogram contig length is shown in Supplementary Figure 4. The rarefaction curve, displayed in Supplementary Figure 5, demonstrates the amount of diversity captured by a given number of reads in a sample.

### 3.2. Comparative analysis of bacterial diversity (SG1 vs. SG2)

**Figure 1:**
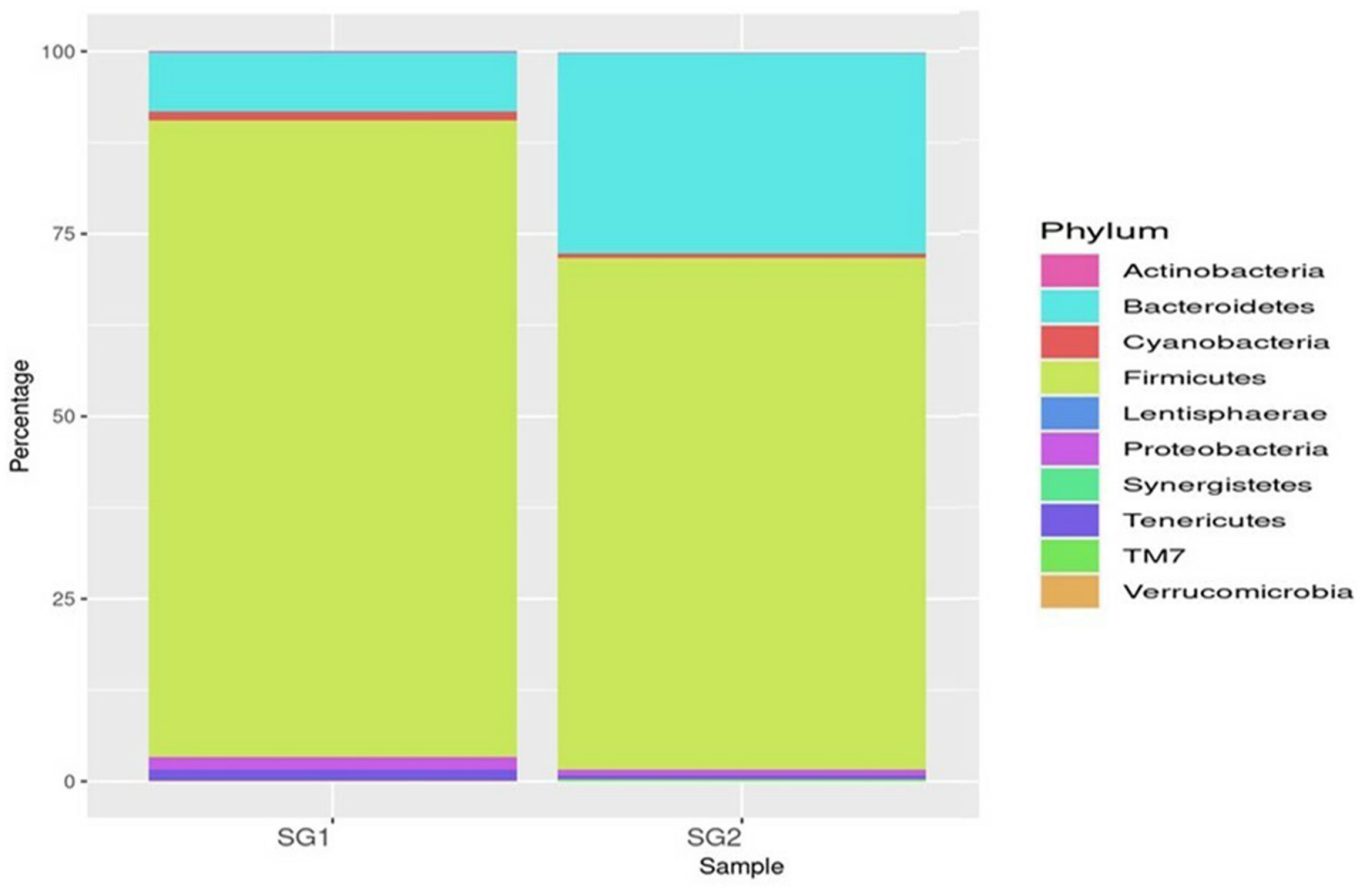
Top 10 phyla.

A total of 333,232 high-quality sequences of the 16S rRNA V4 gene amplicon were obtained from the cecum of the healthy normal group (SG1) and MOLP-treated (SG2) chicken gastrointestinal samples. From each sample, 148,760 and 184,472 effective sequences were obtained, respectively. These effective sequences were clustered into 180 OTUs using a sequence similarity value cutoff of 97% and then taxonomically clustered into 18 phyla, 77 genera, and 39 species (Supplementary Table 3). The bacterial population in the gut cecal sample of the normal basal diet group (SG1) and MOLP group (SG2) chickens revealed a distinctive difference (47%). The microbial population in the ceca of the MOLP-treated group (SG2) exhibited greater diversity of the beneficial bacterial population compared to the normal control basal diet group (SG1). *Bacteroidetes, Firmicutes, Proteobacteria, Cyanobacteria, Tenericutes*, and *Actinobacteria* were found to be the dominant phyla in both samples. The presence of TM7 and *Gracillibacteria* (GN02) was observed in MOLP (SG2) but not in the normal basal diet group (SG1), while the major phyla among both groups were *Clostridia* (97%), *Firmicutes* (85%), *Bacteroidetes* (69%), and others (69%; Supplementary Table 4; Figure 1).

**Figure 2:**
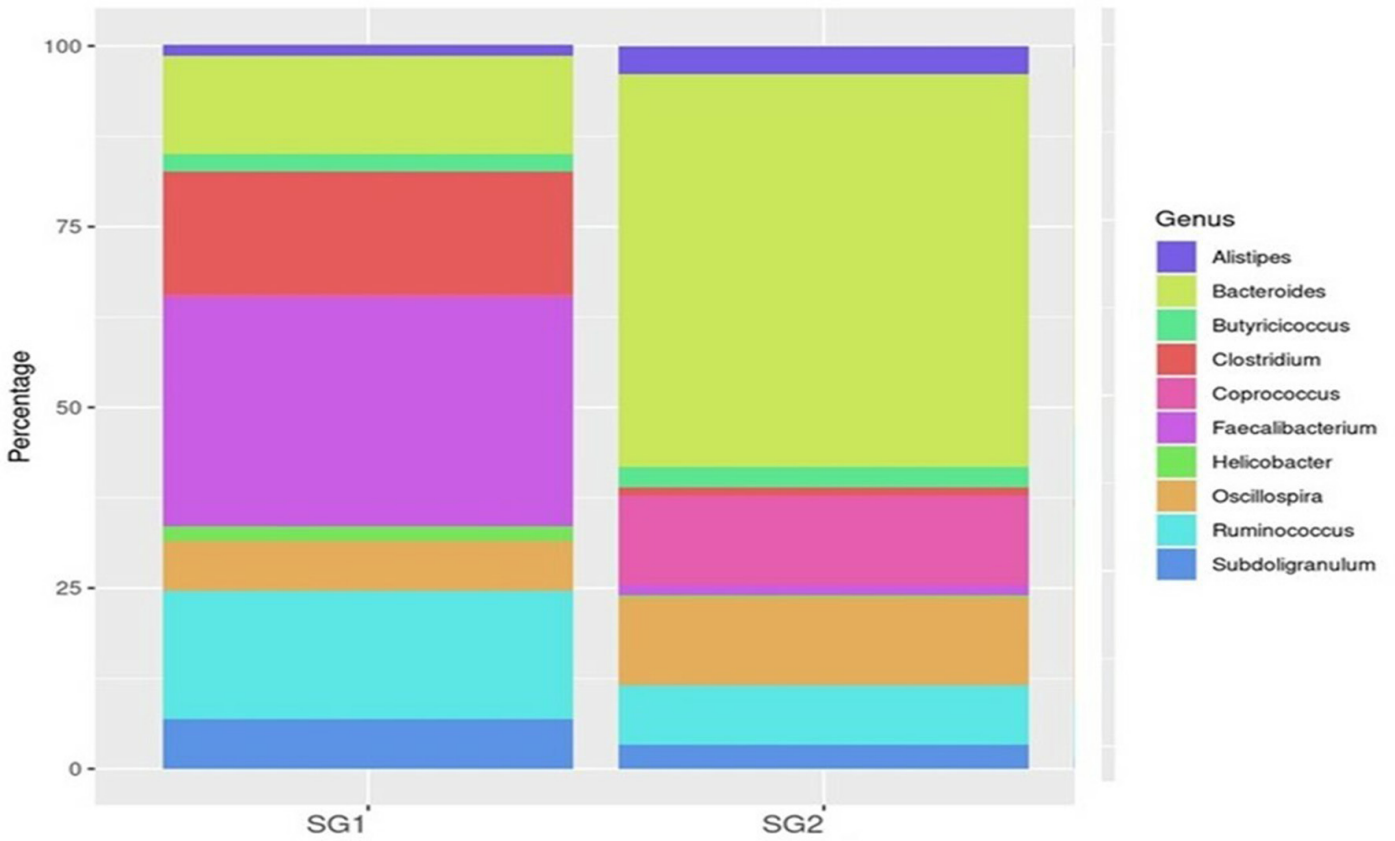
Top 10 genus OTUs distribution.

**Figure 3:**
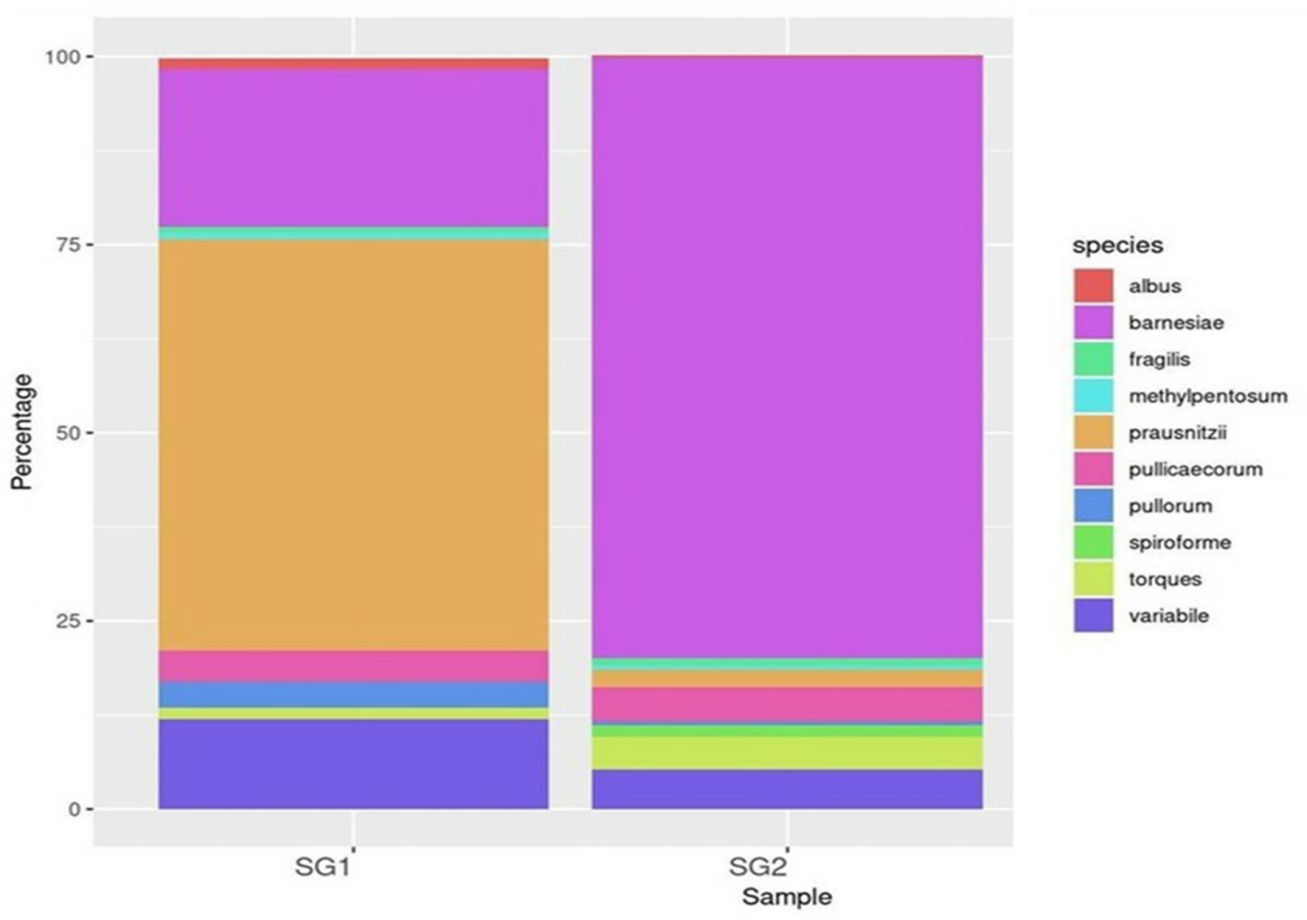
Top 10 species OTUs distribution.

*Bacteroidia* (19 and 80.35%), *Clostridia* (53.5 and 25.21%), and *Epsilonproteobacteria* (86 and 13.2%) were found to be the most predominant genera in the normal basal diet (SG1) and MOLP (SG2) groups, respectively (Figure 2; Supplementary Table 5). There was an observed increase of 61.35% in *Bacteroidetes* and a decrease of 28.3% in *Clostridiales s*pecies in the MOLP-treated group (SG2). The top 10 bacterial species found in the MOLP (SG2) group were *Bacteroides barnesia, Faecalibacterium prausnitzii, Butyricicoccus pullicaecorum, Helicobactr pullorum, Ruminococcus torques, Ruminococcus albus, Clostridium methylpentosum, Clostridium spiroforme, Bacteroides fragilis*, and *Lactobacillus helveticus* (Figure 3).

**Figure 4:**
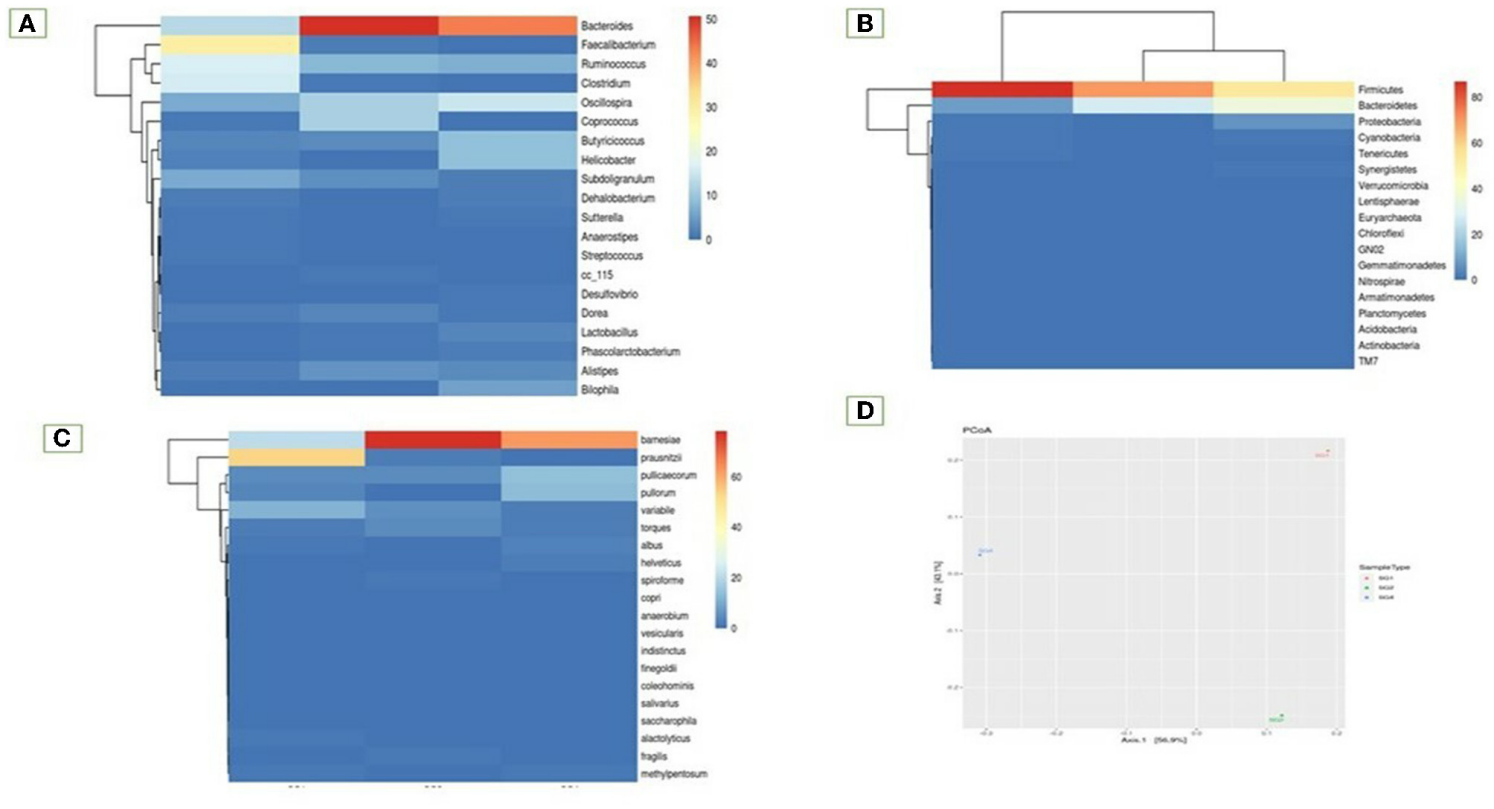
Heatmap analysis of the top 20 phyla **(A)** Phylum, **(B)** Genus, **(C)** Species, **(D)** Principal co-ordinates analysis.

The study also examined species diversity, with *Bacteroides barnesia* and *Bacteroides fragilis* being the major species among the predominant *Bacteroidetes* group, accounting for over 15.3% in the SG1 group and 62.3% in the MOLP (SG2) group, respectively (Supplementary Table 6). The 47% increase in the SG2 group suggests that MOLP may play a role in modulating the gut microbiota of chickens, possibly through bioactive compounds or fiber content (60). However, this requires further research. In contrast to the increase in *Bacteroidetes* in the MOLP-treated group (SG2), a decrease in the species of *Firmicutes*, namely *Ruminococcus torques, Butyricicoccus pullicaecorum*, and *Ruminococcus torques*, from 70.49 to 19.37%, was observed, further supporting the role of MOLP in enhancing beneficial probiotic gut bacteria while suppressing or decreasing pathogenic or non-beneficial bacteria, as shown in Figure 3. This indicates that plant bioactive compounds decreased the *Firmicutes* at the species level by ~51.12%. To analyze the gut microbiome data present in the normal basal diet and MOLP groups, a heatmap analysis was performed, which is a two-dimensional representation of PCA data in which various colors indicate the highest and lowest values in the data matrix while clustering lines or columns of the same value. Heatmap analysis has been used in the visualization of metagenomic analysis. In this study, to analyze the data of the gut microbiome present in the normal basal diet and MOLP group, the data were clustered based on the phyla, genus, and species (Figures 4A–C).

#### 3.2.1. Alpha diversity index

According to the ACE, Shannon, and Inverse Simpson indices, there was a significant increase in the number of species in both the SG1 and SG2 groups. Interestingly, the MOLP-treated group exhibited higher bacterial diversity when compared to the control group. Additionally, it was observed that greater bacterial diversity was associated with increased dominance in the cecum, which is unexpected (Supplementary Figure 6). Despite this, both sites consistently demonstrated increased dominance over time. Upon comparison of the data, it was evident that there was only a small degree of species richness observed within the samples (Supplementary Table 7).

#### 3.2.2. Beta diversity

Fisher's exact test was performed on samples with the help of STAMP to find a statistically significant difference in OTU abundance between the samples (Supplementary Figure 7). Data have been provided in Excel files with the following suffixes: “Fishers-exacttest.xlsx” and “Fishers-exact-test.Significant.xlsx” (*p*-value ≤ 0.05; Supplementary Table 8). Data revealed that 40 species were significant in the MOLP-treated group when compared with the NBD group. TM7 species were unique to the MOLP-treated group but were absent in the NBD group. The Principal Coordinate Analysis (PCoA) is a method for visualizing and analyzing significant differences and identities. According to this analysis, the bacterial population from the MOLP-treated group (SG2) was significantly different from the normal group (SG1). Furthermore, the MOLP group exhibited a decreased count of pathogenic bacteria and an increased population of beneficial bacteria (Figure 4D).

### 3.3. Functional metagenomic analysis

**Figure 5:**
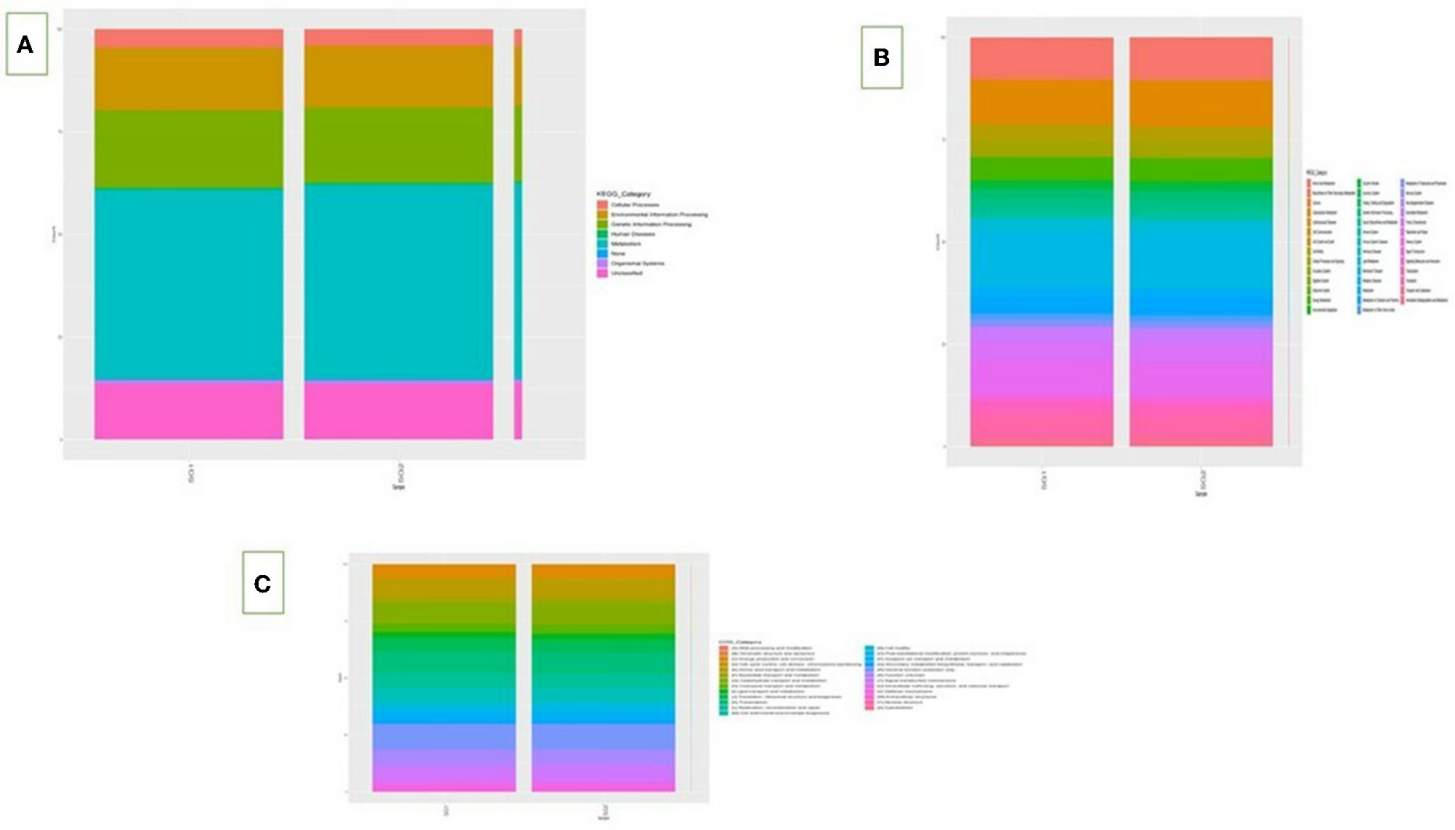
KEGG L1 **(A)** and L2 **(B)** and COG pathway **(C)**.

On the basis of the predicted functional metagenomes analyzed, there were clear differences in the functional activities and effects of the intestinal microflora, including gene pathways related to nutrient utilization such as lipid and carbohydrate metabolism, between the normal basal group and the MOLP-treated group in cecum content. The KO composition showed a distinct difference in the KEGG orthologs between the two groups based on functionality prediction (Supplementary Table 9). Metagenome predictions using the KEGG pathway indicated an increase in the metabolic rate in the lipid and carbohydrate metabolisms for the MOLP-treated group compared to the normal group. Specifically, the presence of 4-nitrophenyl phosphatase [EC:3.1.3.41], which mainly involves microbial metabolism in diverse environments, was 45% higher in the MOLP-treated group. The multiple antibiotic resistance protein also increased by 46.2% in the MOLP-treated group. Moreover, the MFS transporter, DHA1 family, and bicyclomycin/chloramphenicol resistance proteins showed a 33% difference between the normal and MOLP-treated groups (Supplementary Table 10). At the COG level, carbohydrate metabolism ranged between 40.4 and 59.6% in the normal group and the MOLP group, respectively (Supplementary Table 11), showing an increase of 19.2% in the metabolic rate in the MOLP-treated group. Similarly, the lipid transport metabolism rate was 40.6% in the normal group and 59.4% in the MOLP group, revealing an increase of 18.6% compared to the normal group. The metagenome predictions using the COG pathway demonstrated 37.2 and 62.8% beta-galactosidase activity in the normal group and the MOLP group, respectively, with a significant difference of 25.6% between the two groups. This enzyme breaks down lactose into disaccharides to start producing galactose and glucose, which then enter the glycolytic cycle. This enzyme also induces the transgalactosylation of lactose into lactase, which is then cleaved into monosaccharides. Glycosyltransferase, nucleotidyltransferase/DNA polymerase, and aspartate/tyrosine/aromatic aminotransferase enzymes are involved in both lipid and carbohydrate metabolisms and were upregulated up to 59 and 61% in the SG2 group, in contrast to 41 and 39% in the SG1 group. The glycolysis increased by around 22% in the MOLP-treated group (Supplementary Table 12). Note that there was a significant increase of ~20% in the overall metabolism in the MOLP-treated group (Figure 5).

### 3.4. Isolation, identification, and characterization of probiotic bacteria

The cecum content from both the normal basal diet group (SG1) and the MOLP-treated group (SG2) was separately collected and ground with PBS buffer, which was then mashed using a mortar and pestle to obtain a fine paste-like consistency for further analysis. The resulting sample was serially diluted and plated on MRS agar medium and then incubated for 24–48 h under both aerobic and anaerobic conditions (see Supplementary Figure 8). The bacterial isolate was found to grow well in plates supplemented with bile salts and NaCl at low pH, indicating its ability to tolerate extreme conditions such as 6.5% NaCl, 40% bile salt, and pH 9.6. The catalase test revealed the absence of bubbles.

*Enterococcus faecium* was detected in the gut microbial community in the cecal digesta. These bacteria were isolated from the cecum of the control and phytobiotic-treated chickens to obtain the metabolites for further analysis. These isolated bacteria were identified as *E. faecium* and submitted to the NCBI portal with accession numbers OL818307 and ON126220 for the MOLP group and the normal basal diet group, respectively. It was used as an isolate in our study to investigate the production of short-chain fatty acids (SCFA) in response to *Moringa oleifera* supplementation. This study focused solely on the details of *Enterococcus*, despite the identification of several other isolates.

### 3.5. Metabolite isolation, confirmation, and bioassay

While our study focused on identifying and tracking the production of short-chain fatty acids (SCFA) by *E. faecium* in response to MOLP ingestion, it is important to note that MOLP is a complex plant with numerous bioactive compounds. Our study only isolated and analyzed the lipids present in the gut samples, which were of particular interest in relation to SCFA production. However, it is likely that other metabolites of MOLP are also present in the intestine and could potentially have an impact on gut microbiota and health. After isolating the lipids from the *E. faecium* of the two groups, a Sudan black test was conducted to confirm the extraction, and the samples were then stored for subsequent analysis. Results from the Sudan black test indicated that the metabolites obtained from the MOLP group were more potent than those from the normal basal diet group (see Supplementary Figure 9). The extracted lipids were then evaluated for their antibacterial, antioxidant, and anticancer activities. In the future, research can prioritize the discovery and analysis of additional metabolites that are found in the gut of birds after they consume MOLP.

#### 3.5.1. Antibacterial activity and MIC determination

The crude metabolites obtained from *E. faecium* isolated from the MOLP group demonstrated the ability to inhibit the growth of pathogenic bacteria without causing hemolysis, indicating their non-toxic nature. The antibacterial activity of these metabolites was compared with that of those from *E. faecium* isolated from the normal basal diet group against various pathogenic bacteria. The metabolites from *E. faecium* isolated from the MOLP group exhibited higher antibacterial activity against *Klebsiella sp*. (17 ± 1 mm), *E. coli* (19 ± 1 mm), *Acetobacter sp*. (19 ± 1 mm), *Staphylococcus sp*. (21 ± 1 mm), *Enterobacter sp*. (15.5 ± 0.5 mm), *Pseudomonas sp*. (21.5 ± 0.5 mm), and *Proteus sp*. (16.5 ± 1.5 mm) than those from *E. faecium* isolated from the normal basal diet group, which inhibited *Klebsiella* sp. (10.5 ± 1.5 mm), *E. coli* (10.5 ± 1.5 mm), *Acetobacter* sp. (11 ± 1 mm), *Staphylococcus* sp. (17 ± 3 mm), *Enterobacter* sp. (11 ± 1 mm), *Pseudomonas* sp. (10 ± 2 mm), and *Proteus* sp. (11 ± 2 mm), respectively. The isolated metabolites were also compared with standard antibiotics such as penicillin, gentamicin, kanamycin, streptomycin, and vancomycin, which inhibited all pathogenic microorganisms with a 6.5 ± 1.5 to 12.5 ± 0.5 mm zone of inhibition (Table 2). The minimal inhibitory concentration (MIC) of MOLP-treated chicken gut metabolites ranged from 6.5 to 1.75 μL/ml (Supplementary Table 13), with lower MIC values indicating more potent antibacterial activity. The results suggest that MOLP enhances the therapeutic role of the gut microbiota. GC-MS analysis of the metabolites revealed the presence of several short-chain fatty acids, which require further investigation (data not shown here).

**Table 2:** Antibacterial activity of metabolites isolated from both groups tested against pathogenic bacteria.

**Samples**	***Klebsiella*** **spp. (mm)**	***E. coli*** **(mm)**	***Aceto bacter*** **spp. (mm)**	***Staphylococcus*** **spp. (mm)**	***Enterobacter*** **spp. (mm)**	***Pseudomonas*** **spp. (mm)**	***Proteus*** **spp. (mm)**
Penicillin-G	7.5 ± 0.5	9 ± 1	7.5 ± 1.5	6.5 ± 1.5	6.5 ± 0.5	12.5 ± 0.5	7.5 ± 0.5
Gentamicin 10	7.5 ± 0.5	8.5 ± 0.5	8.5 ± 1.5	11.5 ± 0.5	8 ± 1	10 ± 2	11.5 ± 0.5
Kanamycin 30	12.5 ± 0.5	11.5 ± 1.5	12.5 ± 0.5	11.5 ± 0.5	11 ± 1	12.5 ± 0.5	12 ± 0
Streptomycin 10	9.5 ± 0.5	8.5 ± 0.5	8.5 ± 0.5	8.5 ± 0.5	13 ± 0	11.5 ± 0.5	11.5 ± 0.5
Vancomycin 30	-	7.5 ± 0.5	9 ± 1	8 ± 1	11.5 ± 0.5	9.5 ± 0.5	10.5 ± 0.5
NBD SG1	10.5 ± 1.5^a^	10.5 ± 1.5^a^	11 ± 1^a^	17 ± 3^a^	11 ± 1^a^	10 ± 2^a^	11 ± 2^a^
MOLP SG2	17 ± 1^b^	19 ± 1^a^	19 ± 1^a^	21 ± 1^a^	15.5 ± 0.5^a^	21.5 ± 0.5^b^	16.5 ± 1.5^b^

SG1, Normal basal diet without phytobiotic supplementation; SG2-10 g/kg phytobiotic supplementation with normal basal diet; mm, millimeter; the a and b values were found to be significantly different between the two groups.

#### 3.5.2. Antioxidant and anticancer activity

**Figure 6:**
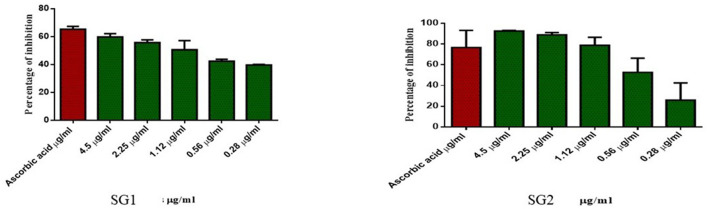
Free radical scavenging activity of various concentrations of SG1 (NBD) and SG2 (MOLP).

**Figure 7:**
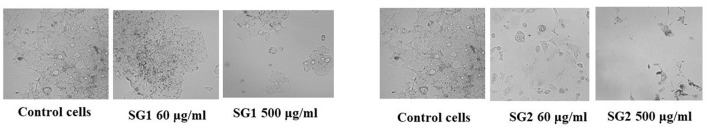
Cytotoxic effect of various concentrations of SG1 (NBDl) and SG2 (MOLP) in comparison with negative control against colorectal cancer (HT-29) cell lines.

Similarly, the antioxidant activity of the metabolite in the MOLP-treated group was higher than that in the NBD group. No toxicity was observed in normal cells, which proves the non-toxic nature of the metabolite and thus suggests its therapeutic role. The metabolite (lipids) isolated from the MOLP-treated group (SG2) exhibited good antioxidant activity (Figure 6) and corresponding anticancer activity against the human colon cancer cell line, HT-29, as shown in Figure 7. These findings suggest that gut metabolites from the MOLP group have the potential to be effective anticancer agents against HT-29 cell lines.

## 4. Discussion

Antibiotic alternatives are employed to decrease bacterial populations and promote growth through numerous processes, including modification and/or suppression of microbial growth, boosting the innate immune system, reducing oxidative stress, and enhancing intestinal integrity (61). Recent scientific discoveries have contributed to the search for effective antibiotic alternatives that provide benefits without promoting resistance. Studies have shown that adding M. oleifera to chicken feed can improve growth, immunity, anticoccidial activity, blood biochemistry (62), antibacterial activity, and antioxidant activity (63–65). This could benefit the poultry industry by improving food safety, health, and economics (66). There are varying opinions on the benefits of *M. oleifera* supplementation in poultry (67), whereas MOLP feed has been reported to improve intestinal permeability and digestive function and favorably modulate the composition of the gut microbiota (68). This could potentially benefit the overall health of the host. Moreover, *M. oleifera* has the potential to be a promising source of nutrients for the development of novel functional foods that could enhance the human immune system through the gut microbiome (69).

Metagenomic analysis has suggested that, when investigating the structure of gut bacterial populations, collecting samples from the cecum is preferable to other intestinal parts for obtaining metagenome sequences (70). This study found that cecum sampling was advantageous for targeting the gut microbial composition as it represented the internal intestinal environment broadly, with greater microbial diversity than other intestinal components (71, 72). Therefore, collecting the cecum for sequencing is preferable for analyzing the activities of the gut microbial community due to the higher level of gene replication activity in cecum samples. The cecal microbiota plays a crucial role in digesting crude dietary fiber, influencing nutrient digestion and absorption in chickens (73). Our metagenomic investigation showed that the gut microbiome was primarily composed of the top 10 phyla, which include Firmicutes, Bacteroidetes, *Proteobacteria, Cyanobacteria, Tenericutes, Verrucomicrobia, Lentisphaerae, Euryarchaeota*, and *Actinobacteria*. Previous studies have reported that Firmicutes and Bacteroidetes are commonly found in mammalian gut samples (74, 75). In the MOLP-treated group, the most common phylum exhibited an increase in Bacteroidia (19 and 80.35%), while the standard baseline diet (SG1 and SG2) showed decreases in Clostridia (53.5 and 25.21%) and Epsilonproteobacteria (86 and 13.2%). The most common genera were *Bacteroidetes* spp., *Faecalibacterium* spp., *Ruminococcus* spp., *Clostridium* spp., *Butyricicoccus* spp., *Lactobacillus* spp., and *Alistipes* spp. The main species identified were *B. barnesiae, F. prausnitzii, B. pullicaecorum, R. torques, C. albus, L. helveticus, C. spiroforme, A. indistinctus, A. finegoldi, L. coleohominis*, and *L. salvarius*. These findings suggest that there was a significant increase in probiotic bacteria in the chicken group supplemented with MOLP. A microbiota dominated by distinct fiber-utilizing microbes could impact immune function by producing varying amounts and types of short-chain fatty acids (SCFAs) using the same carbohydrate substrates (76). According to Xie et al. (77), SCFA production may play a role in maintaining a healthy gut environment (78). Starch is a suitable substrate for the colonic microbiota, and its metabolism depends heavily on the cooperation of Firmicutes and Bacteroidetes. *Bacteroides* spp. and *Lactobacillus* spp. have simple enzyme systems that ferment fructans and lactose, respectively (71, 79). The dominant bacterial diversity in the cecum of CARI-Nirbheek country chickens was found to be *Clostridium* spp., which can impact gut health through interactions with the immune system and the synthesis of metabolites (44). Butyrate is the most versatile of the SCFAs and has been linked to numerous benefits for gut health, including serving as an energy source for colonic epithelial cells, possessing anti-inflammatory properties, reducing luminal pH, limiting bile salt solubility, decreasing ammonia absorption, and preventing pathogen invasion (80). Sowmiya et al. reported that the country chickens had higher bacterial diversity than broiler chickens and that phytobiotic supplementation decreased the amount of Firmicutes.

*Enterococci* spp. are probiotic bacteria that belong to the lactic acid bacteria (LAB) family. They play a vital role in food fermentation and deterioration and are also used as probiotics in both humans and slaughtered animals. It can be found in various environments, such as air, soil, water, and the gastrointestinal tracts of animals and humans (81). As *Enterococci* spp. are associated with the gastrointestinal system, screening them from animal feces and intestines is a common and effective procedure. In recent decades, the beneficial effect of *Enterococci* spp. from animal and human feces in the food and livestock sectors has been widely studied (82). *Enterococcus faecium* is one of the most important *Enterococci* spp. because it is well-adapted to living and thriving in the gut, and it helps to maintain a healthy gut environment by competing with pathogenic bacteria for resources necessary for their survival. It also competes for adhesion sites with hazardous bacteria, which are spots on the cell surface where other cells and chemicals can attach (83). Consequently, this multitasking strain is commonly found in human probiotic supplements. AAFCO approves *E. faecium* for use in animal feed as a direct-fed microbe as long as it is non-toxigenic (84).

The present study isolated *E. faecium* from the intestines of chickens from both SG1 and SG2 groups and examined its form, size, and organization using microscopic analysis. Smooth, mucoid, and white *E. faecium* colonies were observed on agar. During cassava fermentation, the chemicals produced by *E. faecium* were found to be crucial in inhibiting the growth and survival of pathogens, as reported by Ilango and Antony (85). The production of metabolites in this study may be attributed to the inclusion of MOLP in the diet. The MOLP-treated group had a more diverse microbial population and produced more effective metabolites than the chicken-based basal diet group. Lipids are a diverse group of biomolecules that can have various biological activities, including antioxidant and anticancer activity. Specifically, the lipids that we isolated in this study were characterized further beyond their identification, and GCMS analysis revealed these lipids as SCFA. However, previous studies have reported that certain lipids, such as short-chain fatty acids (SCFAs), can possess anticancer (86), antioxidant (42, 43), and antibacterial properties (87, 88). For example, SCFAs have been shown to reduce oxidative stress and inflammation, which are associated with the development of various diseases, including cancer. Additionally, certain lipids, such as omega-3 fatty acids, have been shown to have anti-inflammatory (89) and anti-cancer properties (90, 91).

According to Lombogia et al. (92), the presence of bacteria in the digestive system that produces dominant metabolites helps maintain a balanced environment. Bioactive molecules with potent antibacterial activity against various pathogenic bacteria, including enterotoxigenic *E. coli, Salmonella*, and *Staphylococcus aureus*, were discovered in organisms found in panda feces and fish (93). The current study demonstrated that the isolates obtained from the MOLP-treated group exhibited strong antibiotic activity against uropathogens, such as *Staphylococcus* spp., *E. coli, Acetobacter* sp., *Klebsiella*, and *Pseudomonas* sp., that were isolated from patients with urinary tract infections.

This suggests that gut metabolites from the treated group have the potential to prevent urinary tract infections by altering barrier function, repairing damaged DNA, regulating cell apoptosis, killing target cells, and performing anti-inflammatory activities through signaling pathways. Intracellular extracts from *Lactobacillus* spp. have been found to reduce and scavenge reactive oxygen molecules, as well as chelate metal ligands, thereby exhibiting antioxidative properties (94). *In vitro* studies have also demonstrated that intact LAB cells have antioxidant activity (95), which suggests that consuming LAB-containing meals or supplements is beneficial for health. The intracellular components produced by *Enterococcus* spp. in the gastrointestinal system can also serve as antioxidative agents, utilizing intact molecules as drug carriers across the gastrointestinal tract. The human body and food systems continuously produce a wide range of oxygen-centered free radicals and other reactive oxygen species, which can be mitigated by antioxidative agents (96).

The translocation of *E. faecium* has been associated with colorectal cancer development. Immune defense enhancement and anti-proliferation are two mechanisms for preventing colorectal cancer [CRC; (97)]. Additionally, direct exposure to *Enterococcus* spp. cells resulted in a G2 cell cycle arrest, indicating that commensals, including intestinal microbes, may contribute to cellular transformation and tumorigenesis. *Enterococcus aecalis* has also been linked to various types of colorectal polyps, which are believed to be a common cause of colorectal cancer. In studies with adenomatous polyposis coli mutant mice, the administration of a heat-killed strain of *E. faecalis* EC-12 diminished the development of polyps in the small intestine by suppressing β-catenin signaling (98). Karpiński et al. (99) demonstrated that *E. faecalis* grown on an aggressive colorectal cancer cell line (HCT-116) reduced the expression of the FIAF protein, which is frequently found in different types of cancer. Our research found that the MOLP-treated group of *E. faecium* produced more protective metabolites than the untreated group. Compared to the typical basal diet group, these metabolites had the ability to trigger apoptosis in the HT-29 colorectal cancer cell line. MOLP controlled the amount of Bacteroides during the development of CRC, which is linked to intestinal inflammation. Isolates from the MOLP-treated group induced toxicity with an IC50 value of 57.47 μg/ml, while isolates from the control group induced toxicity with an IC50 value of 163.6 μg/ml. This may be due to the MOLP-treated group's production of useful metabolites.

## 5. Conclusion

Metagenomics has provided new insights into microbiomes and the intricate interactions between microorganisms and their hosts. This study shows that nutrition, particularly phytobiotics such as *Moringa oleifera*, can impact the composition of the intestinal microflora, which is essential for maintaining gut health and enhancing the immune system. Beneficial microbes such as *Bacteroides fragilis, Bacteroides barnesiae, Lactobacillus* spp., and *Butyricoccus pullicaecorum* were found to have higher microbial populations than harmful ones such as *Clostridium species*, underscoring the importance of modulating phytobiotics such as *Moringa oleifera* to improve gut health. The growth of probiotic bacterial populations resulted in an increase in the production of SCFA and other critical metabolites. Metabolites from MOLP-treated chicken gut, particularly SCFA, have demonstrated antibacterial, antioxidant, and anticancer properties. Further investigation is required to explore the underlying mechanisms and pathways. In the near future, the use of phytobiotics to manipulate the gut biota could be a groundbreaking approach for treating various diseases.

## Data availability statement

The datasets presented in this study can be found in online repositories. The names of the repository/repositories and accession number(s) can be found in the article/Supplementary material.

## Ethics statement

The animal study was reviewed and approved by Institutional Animal Ethics Committee (IAEC), Department of Animal Science, Bharathidasan University, Tiruchirappalli-600024; Reg No. 418/GO/Re/S/01/CPCSEA, dt.24.07.2018; (BDU/IAEC/P06/2021). Written informed consent was obtained from the owners for the participation of their animals in this study.

## Author contributions

SS and JS conceived and designed this project, managed the animal breeding, collection of cecum samples, and metagenomics data analysis. ZM, VS, and YG performed the isolation of metabolites. ZM and SS performed the bioassay methods. SS, ZM, JS, and RS wrote the manuscript. JS and RS provided guidance for manuscript drafting and conceptualization. All authors approved the final version of the manuscript.
