# Supplementary material for: Investigating the modulatory effects of Moringa oleifera on the gut microbiota of chicken model through metagenomic approach
Source: Front Vet Sci. 2023 Jun 1;10:1153769. doi: 10.3389/fvets.2023.1153769 (PMC10267347; doi:10.3389/fvets.2023.1153769)
Supplement: Supplementary file 1 [file Data_Sheet_1.docx]

Supplementary Information for:

**Modulatory effects of Phytobiotic, *Moringa oleifera* fed Gut Microbiota in Chicken models and Biological characteristics of the metabolites – A Metagenomics analysis**

**Sowmiya Soundararajan^1^, Jasmine Selvakumar^1*^, Zion Mercy Maria Joseph^1^, Yuvapriya Gopinath^1^, Vaishali Saravanan^1^, Rameshkumar Santhanam^2*^**

**^1^**Department of Biotechnology & Bioinformatics, Bishop Heber College (Autonomous), Affiliated to Bharathidasan University, Tiruchirappalli, TamilNadu, India 620017

^2^Faculty of Science and Marine Environment, Universiti Malaysia Terengganu, Kuala Nerus 21030, Malaysia

*Corresponding author: Jasmine Selvakumar & Rameshkumar Santhanam

E-mail address: [jasminebiotech@bhc.edu.in](mailto:jasminebiotech@bhc.edu.in) & [ramesh@umt.edu.my](mailto:ramesh@umt.edu.my)

**Supplementary Figures**

Supplementary Figure 1. Plant collection to prepare a *Moringa oleifera* leaf powder

Supplementary Figure 2. Experimental design of birds

Supplementary Figure 3. Histogram of reads with average sequence & base quality scores

Supplementary Figure 4. Histogram representing contig length distribution.

Supplementary Figure 5. Rarefaction curve

Supplementary Figure 6. Alpha diversity

Supplementary Figure 7. Beta diversity for bacterial abundance

Supplementary Figure 8. Isolation of probiotic bacteria from chicken gut

Supplementary Figure 9. Isolation of metabolites –Lipids

Supplementary Figure 10. Minimal inhibitory concentration

Supplementary Figure 11. Antioxidant activity

Supplementary Figure 12. Anticancer activity – MTT assay

# Supplementary Tables

Supplementary Table 1. V3V4 Primer sequence used for sequencing

Supplementary Table 2. Read QC Statistics of the sequencing data.

Supplementary Table 3. Raw Operational taxonomic Units from SG1 and SG2 groups

Supplementary Table 4. Phylum abundance distribution of gut metagenomes

Supplementary Table 5. Genus abundance distribution of gut metagenomes

Supplementary Table 6. Species abundance distribution of gut metagenomes .

Supplementary Table 7. Alpha diversity profile

Supplementary Table 8. Beta diversity profile

Supplementary Table 9. Functional Analysis- KEGG category L1 & L2

Supplementary Table 10. Metagenome predictions_ KEGG category

Supplementary Table 11. Functional Analysis – COG category

Supplementary Table 12. Metagenomes predictions_COG category

Supplementary Table 13. Antibacterial activity & Minimal inhibitory concentration

Supplementary Table 14. Antioxidant assay

Supplementary Table 15. Anticancer activity MTT assay


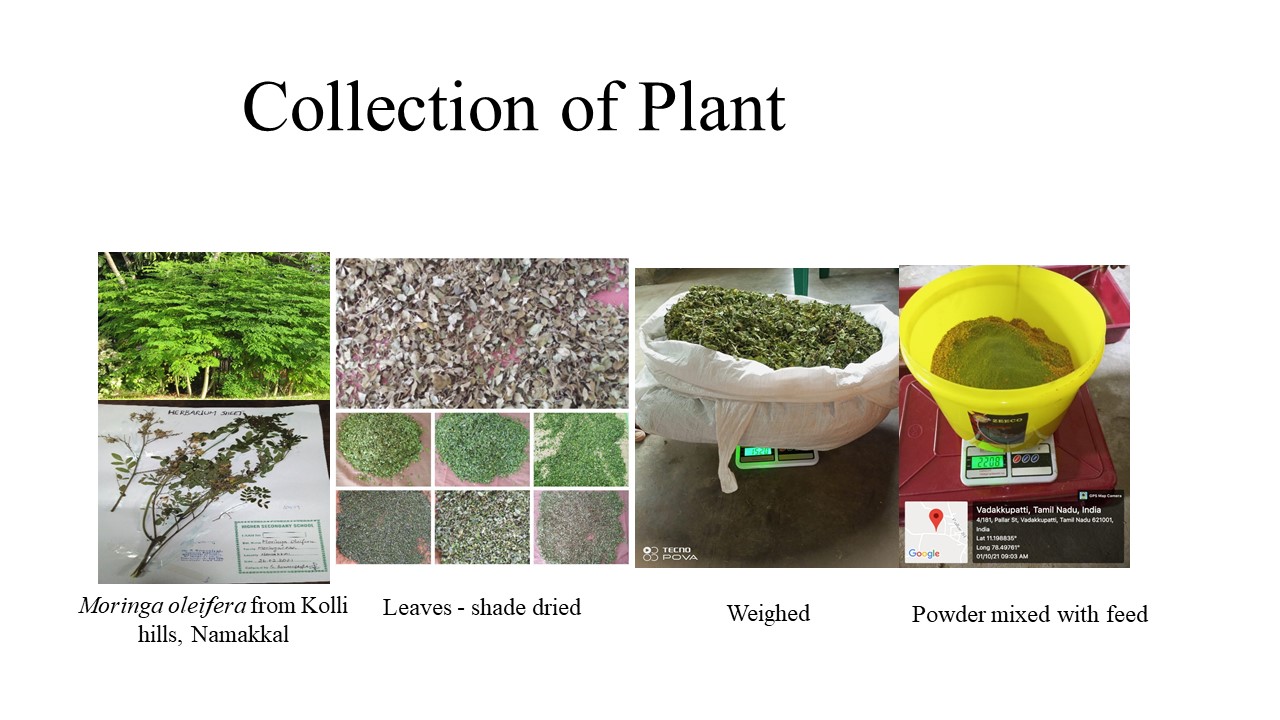


**Supplementary Figure 1: Plant collection to prepare MOLP**


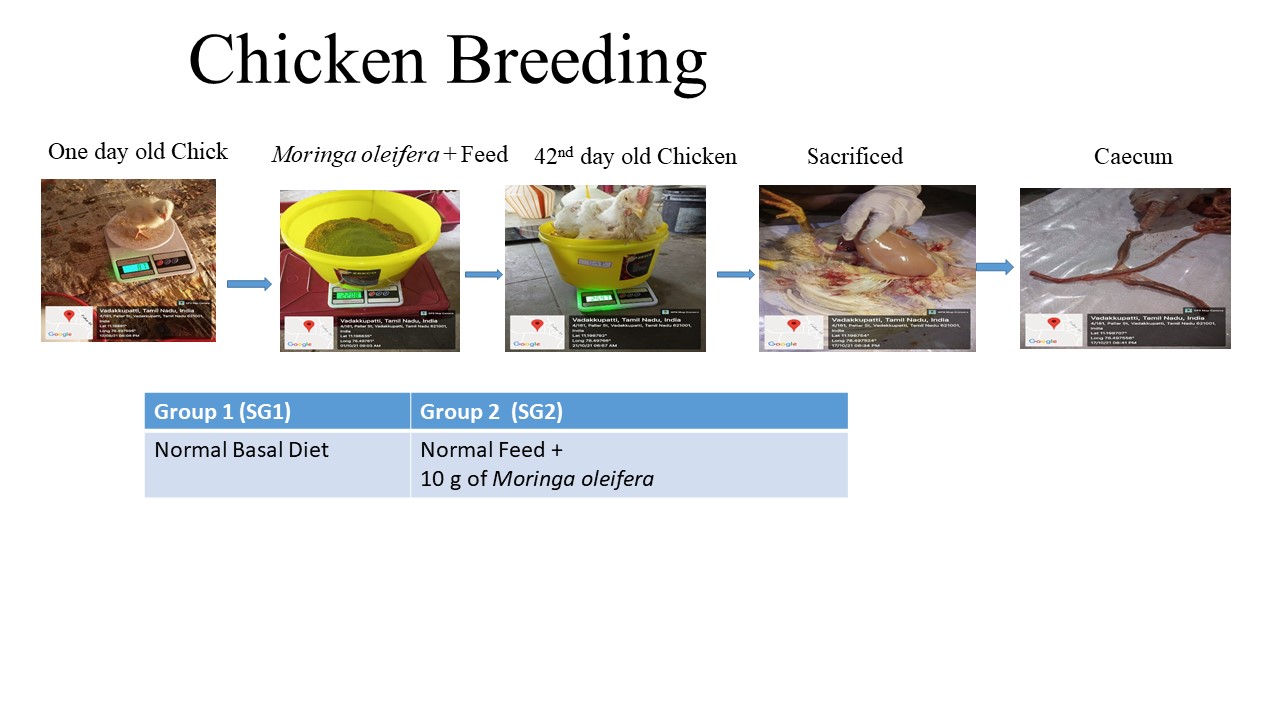


**Supplementary Figure 2: Experimental design of birds**

**
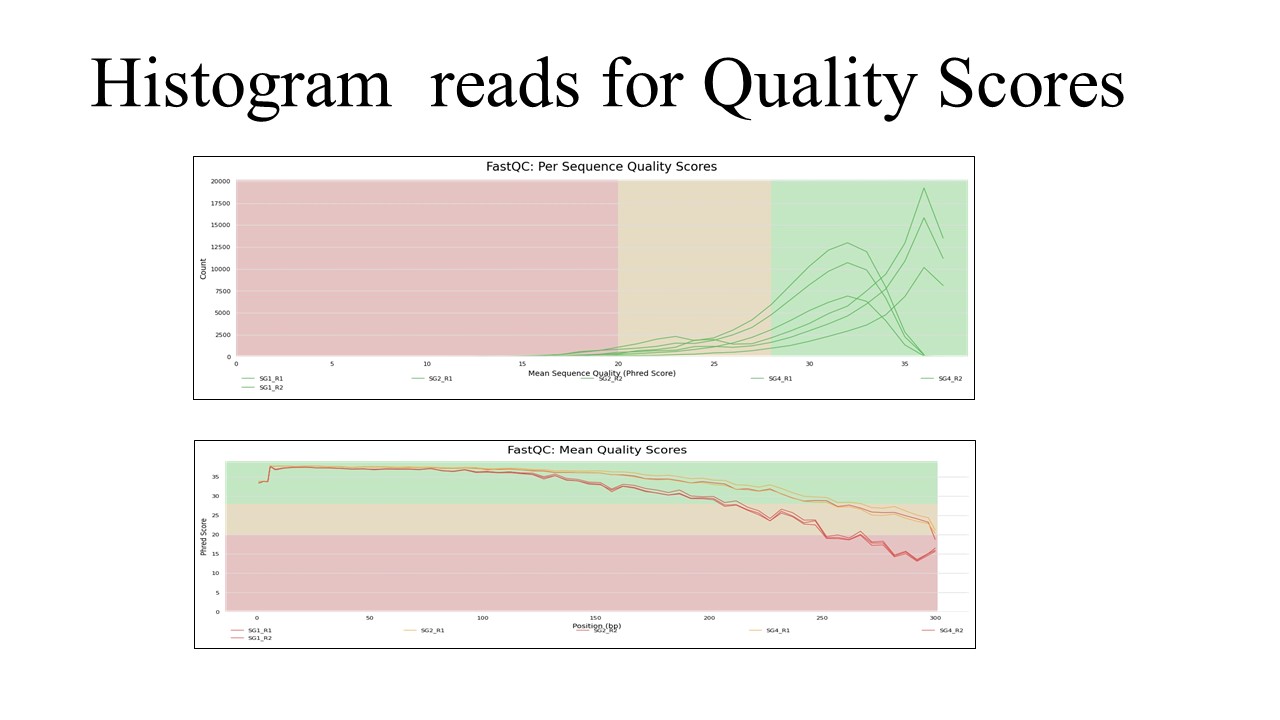
Supplementary Figure 3.** Histogram of reads with average sequence & base quality scores

**
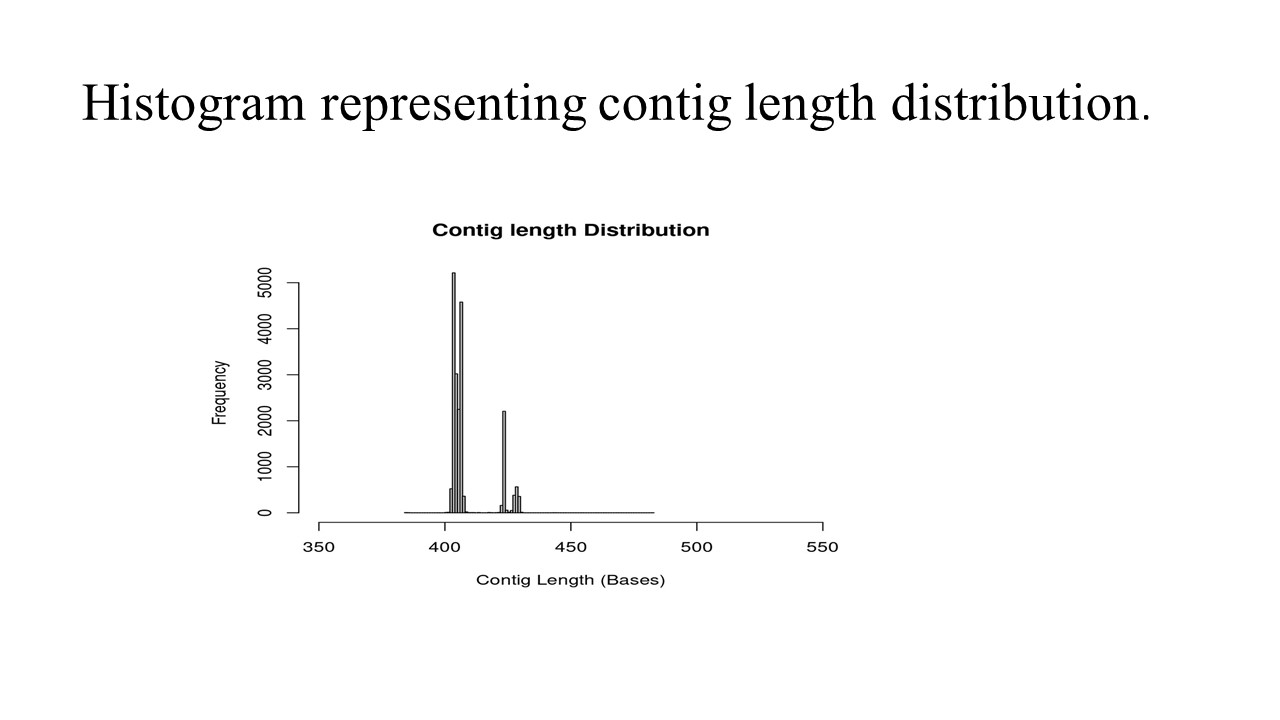
**

**Supplementary Figure 4. Histogram representing contig length distribution**

**
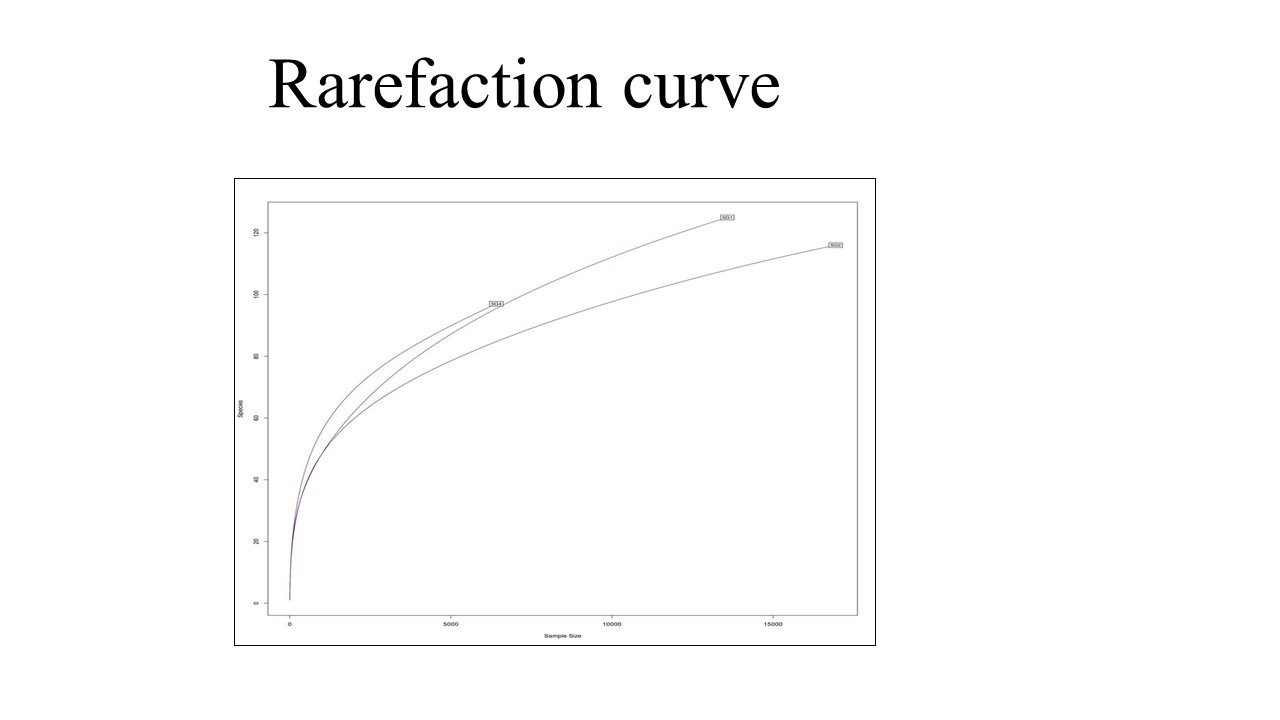
**

**Supplementary Figure 5. Rarefaction curve**

**
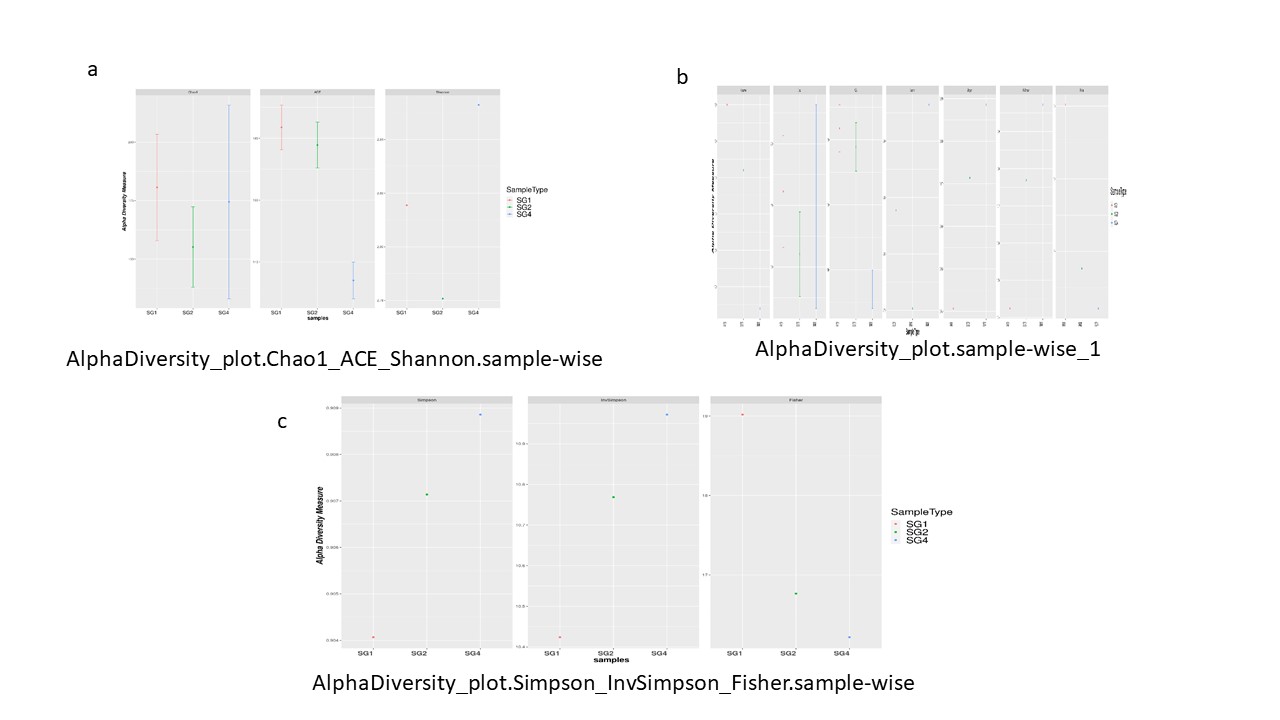
**

**Supplementary Figure 6. Alpha diversity analysis**

**
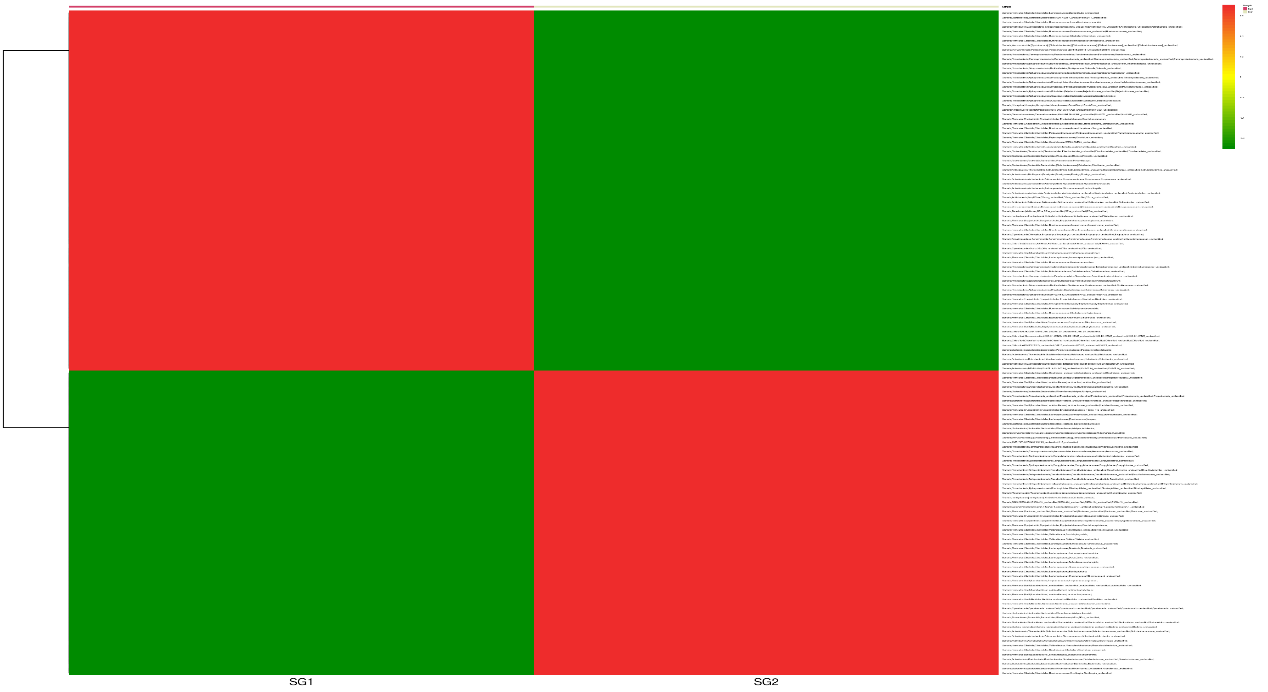
**

**Supplementary Figure 7. Beta diversity**

**
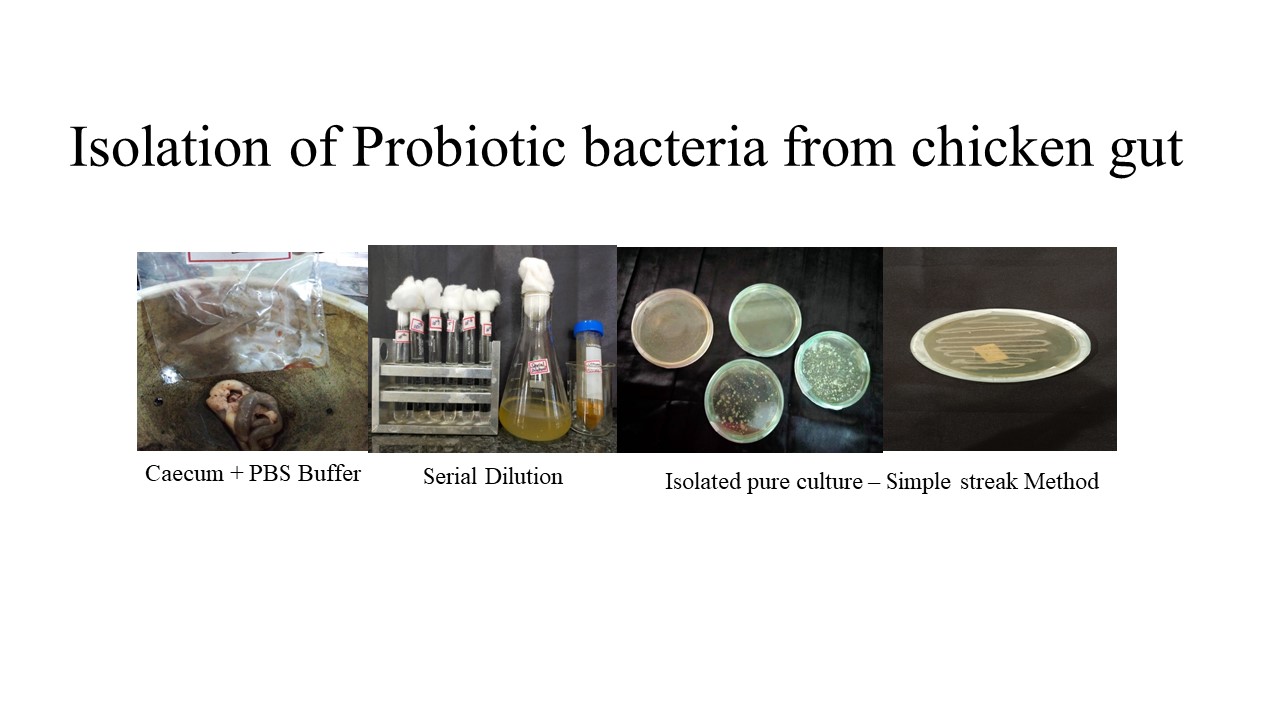
**

**Supplementary Figure 8. Isolation of Probiotic bacteria from chicken gut**

**
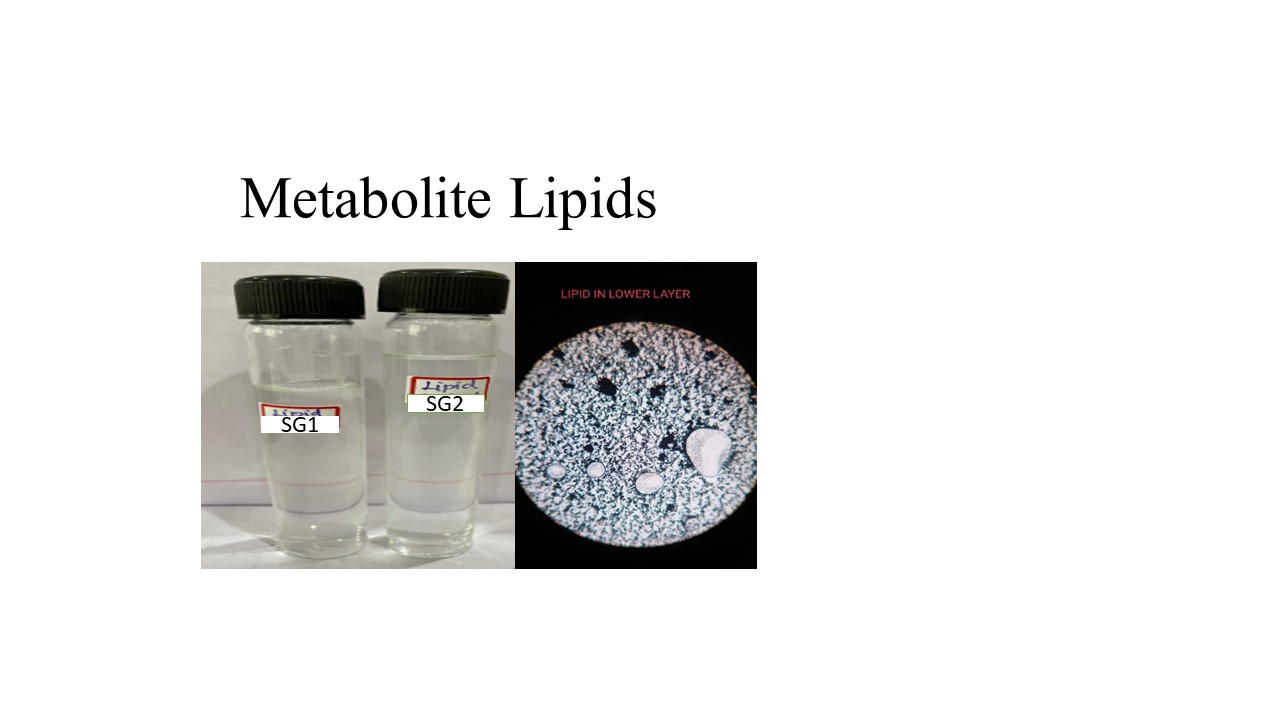
**

**Supplementary Figure 9. Isolation and identification of metabolite lipids**

**
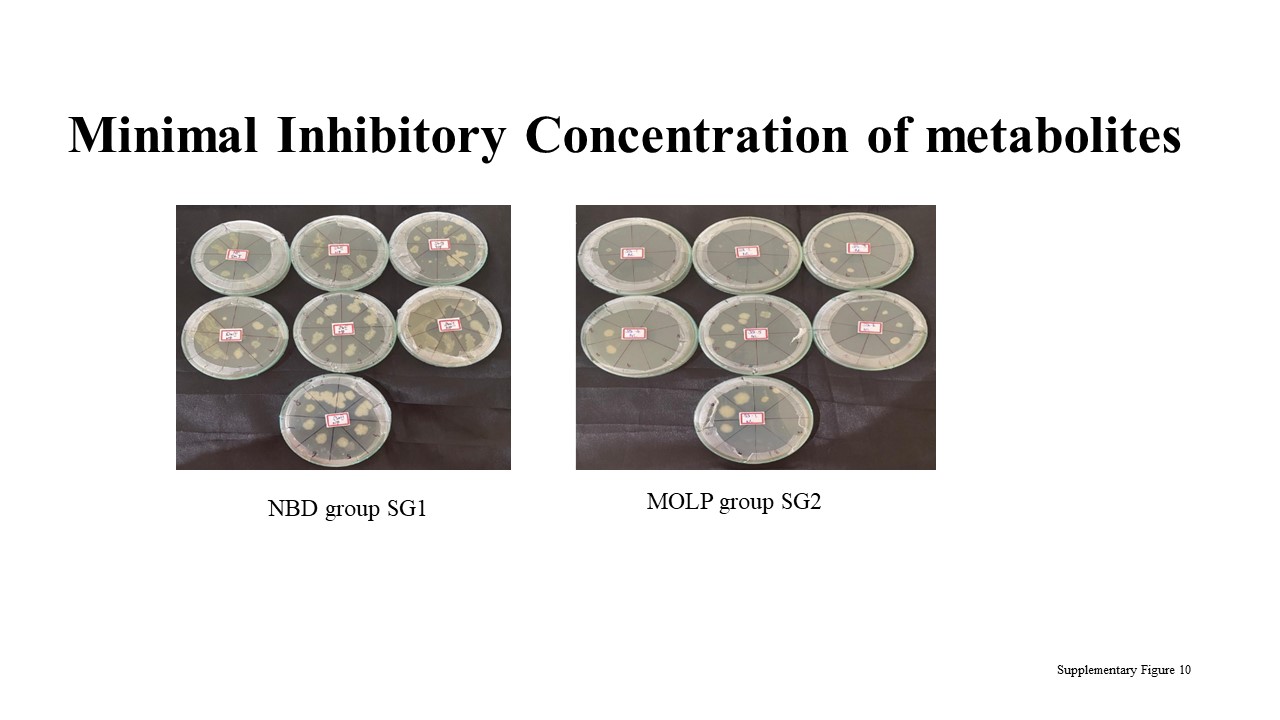
**

**Supplementary Figure 10. Minimal inhibitory Concentration**

**
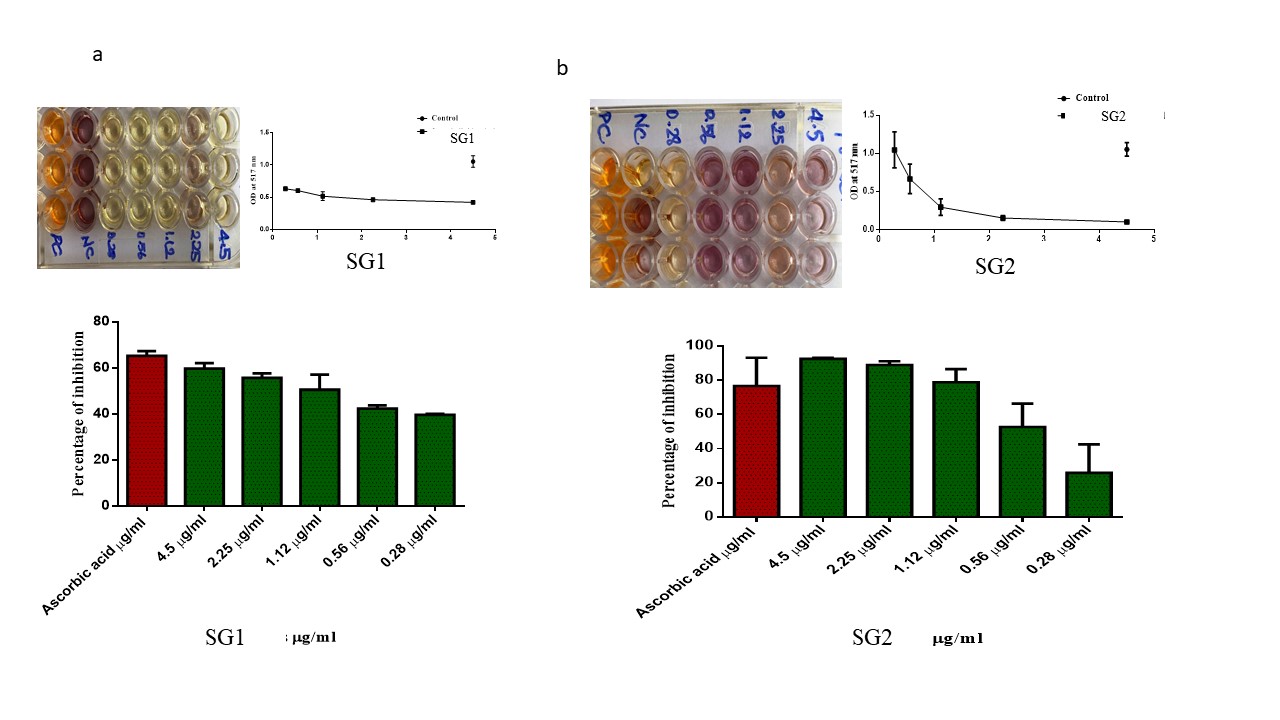
**

**Supplementary Figure 11. Antioxidant activity**

**
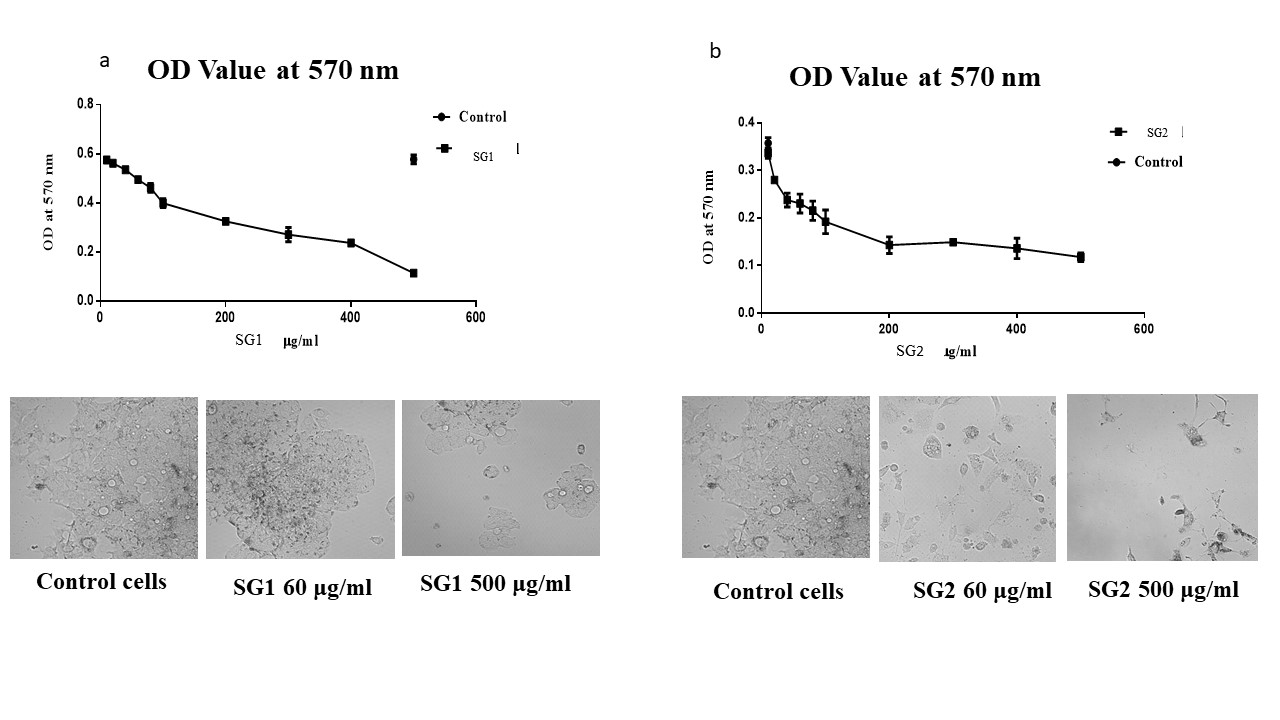
**

**Supplementary Figure 12. Anticancer Activity –MTT Assay a) Normal basal diet group SG1 b) MOLP treated group SG2**
